# Environmental and occupational respiratory diseases – 1053. Plastic bags – a double edge sword

**DOI:** 10.1186/1939-4551-6-S1-P51

**Published:** 2013-04-23

**Authors:** Deepika Ramachandra

**Affiliations:** 1 Department of Microbiology, Bangalore Medical College and Research Institute, Bangalore, India

## Background

Plastic is convenient, lightweight, unbreakable and relatively inexpensive. However, there are both environmental and health risk from the widespread use of plastics. Bisphenol A, a chemical that mimics the action of the human hormone estrogen, can leach from polycarbonate plastic. Bisphenol A has been found to stimulate prostate cancer cells7 and causes breast tissue changes in mice that resemble early stages of breast cancer in both mice and humans. Reusable bags for transport of groceries from the store to the consumer’s home have become popular in recent years. Since these bags are often reused, and used potentially for multiple purposes, the possibility for contamination of food products as well as the consumer’s hands. Most food borne illnesses are believed to originate in the home. Reuse of bags creates an opportunity for cross contamination of foods. Study was to assess the potential for cross contamination of food products from reusable bags used to carry groceries. It is recommended that the public needs to be educated about the proper care of educated about the proper care of reusable bags by printed instructions on the bags or through public service announcements improper cooking or handling of foods. Reusable bags if not properly washed between uses, create the potential for cross contamination of foods.

## Methods

*Type of study*: Cross-sectional study.

*Sample size*: N1=100 from hospital wards,N2=50 from household kitchens.

*Source of data*: This is a cross-sectional study carried out in the Department of Microbiology, Bowring and Lady Curzon Hospital, Bangalore Medical College and Research Institute. The 100 plastics were taken from the wards of the 4 attached hospitals of BMCRI and 50 are taken from household kitchen.

*Inclusion criteria*: Plastics which are used for more than once.

*Exclusions criteria*: Plastics which are used more than once.

## Results

Staphylococcus strains are isolated. Complete results will be given during project submission.

## Conclusions

Plastics apart from being Non Biodegradable Waste is also a Potential Source of Spread of Nosocomial Infections. Hence needs to be banned completely.

